# Healthcare providers and caregivers’ perspectives on factors responsible for persistent malnutrition of under 5 children in Buhweju district, South Western Uganda; a phenomenological qualitative study

**DOI:** 10.1186/s12889-021-11432-1

**Published:** 2021-08-03

**Authors:** Catherine N. Abaasa, Godfrey Zari Rukundo, Savino Ayesiga, Susan Pearl Atukunda, Susan Campisi, Shawna O’Hearn, Noni MacDonald

**Affiliations:** 1 Department of Medical Laboratory Sciences, Mbarara University of Science and Technology, P. O. Box 1410, Mbarara, Uganda; 2 Department of Psychiatry, Mbarara University of Science and Technology, Mbarara, Uganda; 3 Department of Anatomy, Mbarara University of Science and Technology, Mbarara, Uganda; 4 Uganda Industrial Research Institute, Kampala, Uganda; 5 Hospital for Sick Children, Toronto, Canada; 6 Department of Nutritional Sciences, University of Toronto, Toronto, Canada; 7 Global Health, Faculty of Medicine, Dalhousie University, Halifax, Canada; 8 Department of Paediatrics, Faculty of Medicine, Dalhousie University, Halifax, Canada

**Keywords:** Malnutrition, Nutrition, Village health teams, Caregivers, Healthcare providers

## Abstract

**Background:**

Unacceptably high levels of childhood malnutrition have been registered in all regions of Uganda over the years. Buhweju district alone contributed 46% prevalence of childhood malnutrition to the 47.8% estimated national prevalence for the whole of western Uganda in 2014. This study assessed health provider and caregiver opinions on factors responsible for persistent malnutrition among under five children in Engaju and Nyakishana sub counties.

**Methods:**

In this phenomenological qualitative study, we conducted two key informant interviews and six focus group discussions with Village Health Team members and care takers of under five children in Engaju and Nyakishana sub-counties respectively.to explore their opinions on the factors responsible for persistent malnutrition in Buhweju District in May 2018. Data were thematically analyzed manually and using Atals Ti 7.5.

**Results:**

Historical and geographical challenges, poverty and economic occupation, parental alcoholism and domestic violence as well as inadequate childcare services were identified as factors responsible for persistent malnutrition among under five children in Engaju and Nyakishana sub counties.

**Conclusion:**

Persistent malnutrition in under five children is mainly due to historical and geographical challenges and its associated factors that include poverty and economic occupation, parental alcoholism and domestic violence and inadequate childcare services. Thus literacy education for mothers and young adolescent boys and girls through engaging local leaders, local nongovernmental organizations and Companies operating in the district to contribute to social services provision would limit the domestic violence and increase sensitization on male responsibilities in the children care in Buhweju district.

**Supplementary Information:**

The online version contains supplementary material available at 10.1186/s12889-021-11432-1.

## Background

Nutritional wellbeing is fundamental to the attainment of the full social, economic, mental and physical potential of individuals, communities and populations [1–3]. Factors associated with malnutrition happen at local, regional, national and international levels. They range from environmental, social, economic, socio-demographic and political causes that are deeply interrelated, and mutually affect each other [4]. In Uganda, malnutrition has been associated with immediate, basic and underlying factors as well as food prices, poverty and unemployment [5]. Children whose parents engage in agriculture and manual work, peasant farmers or parents employed by non-family members, as well as age of the mother, and age and sex of the child; are at higher risk of stunting and underweight compared to the children from pastoralists’ families [6, 7].

Although all regions of Uganda have registered unacceptably high levels of childhood malnutrition over the years, the distribution has not been even [8]. The western region of Uganda has persistently registered the highest levels of childhood malnutrition, yet, this is the region with plentiful food production and is sometimes referred to as “the food basket” of the country [9, 10]. In the Ankole region of southwest Uganda, children under 5 years 29.3% were stunted, similar to a national average of 29.0% but Buhweju district had stunting levels of 51–55.6% [11, 12]. This study assessed health provider and caregiver opinions on factors responsible for malnutrition among under five children in two sub counties of Engaju and Nyakishana in South West Uganda in May 2018.

## Methods

### Study setting

Buhweju is a rural District in Southwestern Uganda with a population of 124,044, with hilly geographic terrain and hence poor infrastructure including road network [11]. The population is mainly engaged in agriculture practicing subsistence farming and/or working on tea and coffee plantations. Some male members of the community work in the gold mines.

### Recruitment and eligibility of the study participants

This was a phenomenological qualitative study. Caretakers of children aged between 0 to 59 months, as well as the District Health Officer, nutrition focal person (a nurse with nutrition training) and community health workers known as Village Health Team (VHT) members were purposively sampled from communities in Engaju and Nyakishana sub-counties and at the district. The VHT coordinators in these communities assisted in recruitment by identifying caretakers and VHT leaders as they are health gatekeepers to these communities.

The identified participants were approached by the study team who introduced the study to the participants by explaining the study purpose and objectives. Participants were eligible if they were above 18 years of age; and were 1) only one caretaker selected per family of observed malnourished children aged between 0 to 59 months and resided in Engaju and Nyakishana sub counties; 2) VHT members, 3) Nutrition focal person and District Health Officer. Participants who did not meet this criterion were excluded from the study. All those approached met the inclusion criteria and agreed to participate. Two focus group discussions with VHT members, two focus group discussions with caregivers as well as key informant interviews were conducted.

### Interview and focus group discussion procedures

Interview guides for the key informant and focus group discussions explored factors underlying the persistent malnutrition. The interview questions included a) What are the factors responsible for malnutrition of under five children? (*family, community, health care, economic, cultural and political*) b) What have people done at the family level to improve child nutrition? (*Primary, Secondary, Tertiary factors*). These questions were derived specifically from the study objectives to; explore the health provider perceptives and examine caregivers understanding of the underlying factors responsible for persistent malnutrition and establish measures being undertaken by households to improve under five malnutrition in Buhweju district. The interviews and focus group discussions were conducted in Runyankore-Rukiiga, the local language, using interview guides translated into Runyankore-Rukiiga, and back translated into English to ensure that the message was correctly translated. The study tools were first piloted with a nutrition focal person at Mbarara Regional Referral Hospital and caretakers of children 0–59 months of age in Nyamitanga division, Mbarara Municipality, Mbarara district for Key informant interviews and Focus group discussions respectively. Necessary changes were implemented. The interviews and focus group discussions with the participants were conducted at a private location at the convenience of the different participants at the time agreed upon with the investigators.

They were not paid for participating in the study but their transport costs were covered. The interviews were conducted by the investigators and a trained Research Assistant between May and June 2018. The interviews lasted between 60 and 90 min, were audio recorded and field notes taken. Interviews and discussions were conducted until indicative thematic saturation was achieved.

Each audio recorded focus group was comprised of 8–12 male and female participants. Study staff also recorded observations through notes during the focus group discussions.CA did the moderating; SA took notes while NP did the recording of the interviews and discussions.

### Data management and analysis

Audio recordings were listened to by CA, SA and NP every after the day’s work. They were transcribed sequentially on the daily basis by CA, SA and NP which helped in giving a deeper insight into the inquiry during the data collection process inline with the study objectives. The data was translated by Research Assistant (NP) and checked by CA and SA. Data analysis was done through different stages of familiarization with data and dual coding was employed. Two members of the research team (GZR and SA) independently read through the transcripts and identified emerging themes and manually identified corresponding quotes by highlighting them with different colors per theme. Data management from interviews and focus group discussions were analyzed differently and merged in one codebook by incorporating data from audio recordings, verbatim notes and nonverbal observations during the interview and discussion processes. A codebook with sections for parent themes, sub themes, description and illustrative quotes was developed from emerging themes. Twelve emerging themes were identified. The identified themes were compressed into four grouped themes. Indicative thematic analysis was done by analyzing statements from participants, identifying commonalities, developing themes and sub themes. The same data was entered into Atlas Ti 7.5. Using the identified themes, each transcript was re-analyzed to reveal the best corresponding quotes. The same process was done for key informant interviews and focus group discussions data.

## Results

A total of 24 males and 49 females composed the different focus group discussions in the two sub-counties (See Table 1). All participants, both male and female were subsistence farmers though some males and females worked on the tea and coffee plantations and local gold mines. The two [2] key informants were a male district health officer and a female district nutrition focal person. They were all employed and each had worked for the district for more than a year at the time of the study.

Four broad themes (See Table 2) were generated (a) historical and geographical challenges (b) poverty and economic occupation (c) alcoholism and domestic violence (d) inadequate child care services

**Table 1:** Study participants

	Description	Sex	Frequency
**Focus Group Discussions**	Village Health Teams	Males	14
		Females	22
	Caregivers	Males	07
		Females	28
**Key Informant Interviews**	District Health Officer	Male	01
	District Nutrition Focal Person	Female	01
**Sub counties**	Engaju	Males	13
		Females	18
	Nyakishana	Males	10
		Females	30

**Table 2:** Codes, themes and subthemes

Codes	Themes	Sub themes
Lack of nutrition services	Inadequate child care services	Breast feeding practices
Nutrition issues are not given priority they deserve		Barriers to immunization
Poverty		Family planning
Food production and care for children is considered a role for women	Poverty and economic occupation	Not Applicable
Alcoholism		
Domestic violence		
Low level of education	Alcoholism and domestic violence	Not applicable
Location, terrain and access		
Water and sanitation		
Economic Occupation	Geographical and historical challenges	Communities use local herbs/ traditional medicines
Health services availability and Utilization		

### Poverty and economic occupation

Healthcare providers reported limited government funding for nutritional programs (food support, nutrition assessment and deworming programs) in Buhweju district compared to other districts. The communities practice subsistence farming but because of poverty, they often end up selling all the produce leaving families with little to eat. At household level, food theft by men also occurred with the men stealing even the little that was available from their wives anusing their money earned on tea plantation or in the gold mines to buy alcohol.

The women were left to care for their families using money they earned after selling produce and working on tea and coffee plantations. The communities lived in poor housing structures and the sanitation was also very poor. There was a high incidence of diseases ranging from diarrhea, malaria and HIV/Aids that also affected the nutritional status of the vulnerable children. Because of the gold mining in the area, children dropped out of school to work in the mines, where they were underpaid/ exploited by middlemen and the little they were paid was used to buy alcohol, gamble or to play pool. Gold mining also led to high school dropouts (child labor).

*“ ideally people are cultivators but with this development that has come in with these murram roads that have been extended to villages, all food is sold off; even today as I was coming I met a full lorry carrying matooke (bananas) to Bwizibwera trading centre because they need money and don’t spare anything for themselves” Healthcare provider-KII.*

“*our men don’t want to support us, their role is going to the bar to drink and stealing our crops after harvesting and you find that you have no lunch and supper for our children and that leads to malnutrition” FGD- Caregivers*

*… … “ there are few people working for the government so when we fix our eyes on men our children will die of malnutrition because men don’t care” FGD-VHTs.*

Having to focus mainly on cash crops, not food crops, takes so much of their time there is little time left to prepare food for their families

“*We have seasonal income because for us we depend on tea and coffee and after harvesting like 3bags of coffee and given like 700000 UGX shillings, you don’t know how the man spent it he can come home with like 200000 shillings and he tells you to put it back in coffee and he tells you that that’s the only money he got and you find that you need school fees and find that you have no balance for feeding the children so they fail to plan for the children because that money is not enough*” *FGD-Caregivers*.

Subsistence farming was not always successful due to poor yields, available land not being very fertile, lack of enough land for cultivation, and limited crops chosen to cultivate so no greens to feed on and no chicken for eggs.

*“Lack of farming space, because when one has enough land, he /she can rear chicken, cows as well as plant some greens and grow enough food for home consumption and sell for school fees but when one doesn’t have land, our children end up being malnourished” FGD-Caregivers*.

### Alcoholism and domestic violence

Men were not seen as supportive with most caregivers and VHTs reporting alcohol abuse and high rates of domestic violence. Women lived in constant fear leading to limited food production and psychological distress impacting their ability to care and feed their children. When men returned home drunk, a usual condition, they ate the food the mother had prepared for the children. These children were left hungry and malnourished. What they earned from commercial agriculture (tea plantations) was not allocated to buying food but was used by men to buy alcohol while the women often bought dresses. Men also spent money on playing pool and gambling.

*“If I had money, I would prefer having local food available because if you have money and you prefer going to drink alcohol and buying expensive dresses, it’s not good because I interacted with mothers and they were like; we can’t buy food when I dig from morning to evening and the man takes everything. If he gives me 100k I would also go and buy a new dress I can’t buy food” Healthcare provider* -KII.

“*Domestic violence! Whereby the man beats the wife and she runs to sleep in the bush and men don’t have time for children because for them in the morning they must go to the bar and no one to cook for them so the child will eat whatever he /she finds because you find that a woman sleeps only for two days in the house and five days sleeps outside and even when she is at home, she has to hurry before the husband comes and pours away the food. Even when they fight at night, the next morning the man goes to the bar with the remaining beans to exchange them for a drink and he leaves the wife and children with nothing to eat so this causes hunger hence leading to malnutrition among children because the food they eat is not good for them*” *FGD-VHTs*

### Inadequate child care services

Malnutrition was associated with a number of factors that were reflective of inadequate health care services including inadequate nutritional services, low levels of immunization, lack of adequate family planning, lack of childcare knowledge and inadequate treatment modalities available. There was lack of adequate implementation of general nutrition services as shown by the low availability of health workers and few health centers and consequently mothers required to travel long distances (mostly on foot), to seek assessment and treatment of nutritional related conditions. Due to the limited capacity of the Health Centers, only nutritional assessment and counseling were offered, and cases of severe acute malnutrition (SAM) were referred to hospitals several miles away without additional support to ensure that the child reached there and received the necessary care. The VHTs lacked sustainable funding to help them complement the inadequate health care human resource. Local leaders were not cooperative in assisting with implementation of known community based nutrition programs and this further impeded the VHTs potential to complement the implementation capacity of nutrition program.

“*We do referral and counselling … it’s the only thing we offer so we counsel the mother on what the child needs to eat and if the child doesn’t need referral they get back to the community. For those who need to be referred, we do.” Health care provider-KII*

*Our leaders don’t cooperate with us but their role is to approve referral by putting a stamp on documents, but sometimes they refuse to approve referral and even when you call them for certain health talks, they refuse and yet people would listen to them than us FGD -VHTs.*

Caregivers in Nyakishana and Engaju reported feeding their children on only a few foods; mainly matooke (type of bananas) and a few times with dodo (greens). This food was limited in access, quality and quantity since most of what is cultivated was sold to raise money to meet other family needs. The banana plantations were destroyed by banana wilt disease. On other occasions, some caregivers did buy posho (Maize meal), sweet potatoes and cassava which their children fed on without sauce.

*… … … … Poor feeding e.g. like feeding on a single type of food and eating each and every food is not good for babies for example matooke (Bananas) without beans but salt and dodo (greens) (FGD-Caregivers).*

There was limited supply of protein rich foods like milk and eggs since very few families owned cows or chicken. A few households fed their children on silver fish and soybean but this was seasonal, expensive and could often only be purchased at markets several kilometers by foot away from their homes.

*… ..“apart from our demonstration gardens which we are trying to develop, you rarely find a vegetable garden at home, they don’t rear animals, you find a goat in a few homes, there are no farms where you will get milk because I have never seen a farm in this district yet these children need milk because they need all these things that will help them get better nutrition” Healthcare provider, KII.*

Caregivers reported to be working for long hours in their gardens and tea farms that were very far from their homes. They leave early and return late when they are tired leading to a lack of time and willingness to prepare meals. Consequently, they prepare meals in large quantities with the intention that the leftover food is to be consumed the following day.

*… … “Limited time for the children whereby you have to wake up very early in the morning going to the garden and you find that you have no time to prepare breakfast for children and even sometimes they have nothing at home to prepare for lunch so they just depend on left overs” (FGD-Caregivers)*

Village Health Teams and Caregivers reported poor breastfeeding practices. Mothers lacked knowledge of proper breastfeeding techniques. Mothers worked on family gardens far from home for long hours and returned home late leaving no time to breastfeed their children. The children are weaned early due to lack of time for breastfeeding, fluctuations in breast milk due to poorly fed mothers.

*“ … … … . You find a mother of a six months’ child breastfeeding the baby right from the garden with unwashed hands while doing other chores and the baby is feeding like a cow” FGD-VHTs.*

*“ … … … ..Sometimes we have no time for our children because we were advised to breastfeed them at least 8 times a day but when you’re busy digging on a family garden, you can’t get time to breastfeed for all that period so by the time you go back home like at 2 pm, the child is already hungry and this leads to malnutrition” FGD-Caregivers.*

Village Health Teams reported that caregivers donot know the purpose of immunization, so they end up forgetting the immunization days. Deworming medication is given at immunizations. Sometimes when they go, the vaccines and deworming medications were not enough to cover all the children. The caregivers claimed that with poor feeding, the vaccines were not important for their children. Due to so many demands on mothers, like digging from distant gardens, cooking and taking care of other family members, immunization and deworming were often not prioritized. Community leaders like pastors also preached against immunization hence making their followers shun immunization and hence did not receive deworming.

*Sometimes we are not informed about immunization days so we end up missing immunization and deworming of our children but there is a VHT who normally moves around giving medicine to our children but if we did not have him our children wouldn’t get immunized.*

*Even some religions don’t support polio immunization like one pastor said I can’t immunize my child against polio and said he never treated any of his children but they are all fine. (FGD VHTs)*

Caregivers reported a lack of access and knowledge on the available family planning services. Caregivers reported not using family planning due to many misconceptions. They believed that family planning affects their health negatively by impacting on the mothers’ ability to do their routine work like digging and household chores. Since most women were the primary source of income for their families, they forewent family planning.

On the contrary, mothers who were willing to use family planning were unable to get the services from their nearby health units because of shortages and limited supplies.

*… … … ..Production of many children. like having 6 children in a compound of almost the same age bracket due to fear of family planning because they say when you’re on family planning, you’re not supposed to overwork and yet when you’re the one taking care of the entire family, you decide to leave it so as to be able to continue working hence leading to many children and that leads to malnutrition due to lack of enough food; they can even be 20 children (that’s how we think) FGD- Caregivers.*

### Historical and geographical challenges

Buhweju was an underserved county in greater Bushenyi district. The district lacks enough public services such as road network and has few health facilities to serve a population of 124,044 people (UBOS, 2014). The education level is low with the majority of the people not educated.

*“There is a problem of education whereby youths don’t want to go to school all they do is waking up very early and go to play pool and whatever you cooked for your children, they will come at night and they eat and sometimes you find that you have your crops in the store. The youths and men steal them so as to get money to play the pool and buy alcohol” FGD -VHT.*

There is poor road network hence transport is poor. There were underserved areas, some without health centers and even when accessed the centres lacked medicines. There was a lack of health facilities, only one Health Centre for the whole sub-county. There was only one Medical Officer in the entire district despite the fact that according to Uganda Health Policy there should be health services provision starting at local council/village level. There was limited interaction between the health workers and community members. There were a limited number of health workers all of whom felt overworked and overwhelmed leading to no time to spend on educating the community.

*“We do what is within our level and healthy facility, we do a lot of referrals since health centres are inadequate to serve and cover the demands of the population and we have realized that nutrition which can be handled at different levels of the facilities can only be handled at Health Centre 111, Health Centre 1V while the lower facilities which are very few only do assessments” Healthcare provider -KII.*

*“If I compare those sub-counties that consistently remain in red in acute malnutrition they are underserved areas and there are some without health centers and access to them is not easy like if you went to Engaju the furthest, you would have appreciated. They* travel *more than about 10kms to access a health center. Even the few staff that are there, they are overwhelmed, they don’t have time to interact with those individuals and educate them” Healthcare provider -KII.*

*“ … … .. in Buhweju we have a thing of witchcraft (mahembe) so you realize that the community is in that tradition and it's blindfolds them instead of fighting against malnutrition, they are looking for who to help them in witchcraft and by the time they go to the facility, the health workers discover that it’s malnutrition” FGD- VHTs.*

*“… … … … I realized that there is a knowledge gap within the community because we expect the community to take some of these things but they seem not to because they think that this issue doesn’t need to get to the health facility. I am worried in the community there are very many children we are losing since the entire community including leaders don’t have enough knowledge about nutrition” Healthcare provider -KII.* There was a lack of political will at low-level councils to advocate for change. Political leaders were money minded. This led to selective donations from the government based on political orientations for example provision of seeds. Social services from the government did not reach the communities as that money was taken by local leaders

*“In Buhweju we need political will which isn’t there at all levels, there are gaps that’s why we came up with multi-sectoral approach and we brought in political leaders and we try to train them and engage them into nutrition and HIV to get to know the situation and they try to push and see some of the issues and gaps that need to be covered but not leaving it to DHOs” Healthcare provider -KII.*

“*Sometimes we hear that the government has sent certain support but we don’t see such support but we think others get or sometimes they bring like beans and they give like 3 people who supported them and they leave others behind” FGD-Caregivers*

Due to the poor terrain, constructing of latrines was hard. The sanitation was poor with sanitation in Engaju sub-county at 43%. The lack of latrines increased the risk of dysentery as did the lack of water for handwashing with the few available latrine structures.

“*There are no systems within the sub-counties to make sure the issue of nutrition and hygiene are followed and the issue of waste management. The health assistant within a sub-county usually visits these communities to make sure these activities are done and does healthy education but we realised that all these systems are not there” Healthcare provider -KII.*

## Discussion

This study explored the perspectives of healthcare providers and caregivers on the factors responsible for persistent malnutrition of under five children in Buhweju district, rural south western Uganda. We found out that factors contributing to persistent malnutrition in the district were multifactorial ranging from socio economic, political, community cultural background and demographic factors and are; historical and geographical challenges, poverty and economic occupation, alcoholism, domestic violence and inadequate childcare services.

Buhweju district, formerly a county of Bushenyi district was an underserved county, due to a hilly geographical terrain, and poor road network hence poor social services. The hilly terrain was found to be the major historical and geographical challenge leading to poor road network hence poor access to social services needed to meet community requirements. This relates to findings by Yourkavitch and colleagues who found differences in health indicators in different geographic areas, and suggested that in order to improve health, challenges in terrain; infrastructure such as road network and sanitation should be addressed to reduce health inequalities [13]**.** There is need to invest in improving roads and infrastructure to enable good food saturation and availability in Uganda [14]. This would improve access to markets, healthcare facilities and communal education especially for women and young people in rural communities positively impacting nutrition of children. Equity focused approaches are also cost effective in improving intervention coverage that result in sharp decreases in child mortality and malnutrition [15]. In addition, there were poor sanitary conditions especially poor toilet coverage, poorly constructed pit latrines and poor access to water since people have to walk long distances to access water. Limited access to water equally impacts on the hygiene of communities. Similarly, a study by Laura Wrisdale and colleagues in Rural South Africa urged that access to water should be considered part of an occupational right which is an integral step in ensuring that water supplies are improved to support better livelihoods inorder to achieve economic and social empowerment, and quality of life for all [16] Most families did not have enough food due to poverty, shortage of land and households especially, males engaging in goldmining, tea and coffee plantations. The workers are exploited by middlemen hence earning less to cater for their household needs and sometimes what is earned is spent on alcohol. This is in line with what has been reported by previous studies which pointed out that unequal distribution of income has an effect on health indicators geared towards improving health since under nutrition among infants living in impoverished rural settings, is associated with poverty and low dietary diversity [17, 18]. The interconnections between malnutrition, poverty, and chronic diseases in Uganda indicates that each of the factors influences the presence and permanence of the other, resulting in a synergistic impact [19]. A study by Bekele and colleagues in Eastern and Western Uganda, suggested that the cost of food items were major barriers to trying new ways of improving their children’s nutrition [20]. To improve the nutrition status in Uganda there is need to improve the economy which would translate to improved income and standard of living since increase in household income leads to increase in food availability, education especially of girls, improvement in the status of women and the quality of the health environment along with the added investment in child nutrition [21]. In contrast some studies have reported that behaviour change in terms of the quality and quantity of care provision, micronutrient supplementation, food fortification and diet supplementation for pregnant women that are essential to reducing under nutrition don’t solely rely on increase in income [22]. Other factors such as maternal depression has a strong negative impact on child nutrition and development since depressed mothers provide inadequate care and diets for their children [23]. There is need to support conditions that strengthen nutrition goals and actions that optimize women’s nutrition, time, physical and mental wellbeing as well as women empowerment [24].

Alcoholism and domestic violence is responsible for persistent malnutrition of under five children in Buhweju. The communities in Buhweju are engaged in Agriculture, both men and women work in Tea and coffee plantations. The funds meant for home use are spent on alcohol hence a setting of domestic violence and its psychosocial effects. Our findings compare with those of Yount and colleagues [25] who highlighted that domestic violence affects early childhood growth and nutrition [25, 26]. A study by Sarah Khan and Stephen Klasen on female employment and spousal abuse in developing countries found out that women working in Agricultural occupations experience more marital abuse [27]. Mothers who experience domestic violence are more likely to have malnourished children [28]. Other studies showed that children of alcoholics are more likely to be malnourished and at high risk for physical abuse [29].

Child malnutrition in Buhweju district was associated with inadequate childcare services and low levels of maternal knowledge on child care rearing practices. The caregivers lack knowledge and skill of nutrition care giving and little has been done to empower these caregivers with knowledge and skills to care for their children but afew get involved. The local leaders havenot helped much in getting the communities to fully participate in the government organized health education trainings or sometimes communication to these communities is very poor due to difference in political affiliations. An earlier study in Buhweju district by Tumwesigye and colleagues (2016) reported that low level knowledge was one of the main predictors of childhood malnutrition. A study in a nutrition education program and community comparison group in Uganda noted that, maternal personal - factors shaped their ability to leverage resources to provide care to their children especially child nutrition, hygiene, access to health care and general household sanitation [30]. In another study in Northern Uganda by Mukunya and colleagues, reported an association of the prevalence of under nutrition, wasting and stunting with rural residence and limited caregiver knowledge of best practices in terms of Integrated Management of Childhood Illnesses (C-IMCI) [31].

### Policy implications

Whereas the Ugandan Ministry of Health has policies to reduce malnutrition in Children, like Immunization, growth monitoring, deworming and Vitamin A supplementation, we suggest that more should be done in implementing these policies to address the persistent malnutrition in Buhweju district. The nationwide interventions alone may not be appropriate for Buhweju due to the unique challenges. Community multisectoral interventions including political, health, nutrition, literacy education for mothers through engaging local leaders, local nongovernmental organizations and Companies operating in the district like the Gold mining companies, tea and coffee companies to contribute to social services provision like, offering scholarships, constructing roads, schools and health facilities as a way of giving back to the community would be a good step towards reducing under five malnutrition in Buhweju district.

The factors highlighted such as domestic violence, alcoholism, low levels of education, sanitation and cultural beliefs must be addressed by local leaders through enacting by laws to help the community comply with set guidelines at national level like working hours for bars, ensuring that every homestead has a latrine, all school going children are in school, emphasizing girl child education and community based education programs in communities through community gatherings, fostering rural cooperatives and income generation schemes, churches and media and making sure that these rules are enforced and community members fully participate.

The observation that members of the communities already have insight into the factors suggests fertile ground for factor related interventions. For example, ensuring that immunization and deworming are seen as important and that these are both available when the mothers bring their children to the health centres; educating mothers on how best to ensure a balanced diet; practical instruction on feeding methods, environmental sanitation, promotion of home grown vegetables reinforcement of the growth monitoring program and working with elders and village leaders to decrease acceptance and perceptions of alcoholism and domestic violence as well as enhancing the role and importance of fathers in providing for their children has potential for significant impact at the grassroots.

### Strengths and limitations of the study

The results of this study reflect the views and opinions of the Healthcare providers and caretakers’ of under five children in Buhweju district. The agreement in their views and opinions is the key strength of this study. The other strength is our ability to work with key stakeholders with respect to childhood nutrition in rural communities like communities of Buhweju district. Since our key informants were employees of Buhweju district local government, this may have acted against us as they may have feared to fully express their views and opinions for the fear of that their employer may access this information. During the informed consent process, we assured all our participants of anonymization and confidentiality. We acknowledge that this study presents views and opinions of caretakers and Village Health Team members in the two sub counties of Buhweju district, we may be missing the experiences of the same in the other eleven [10] sub counties who were not selected, this reflects the views and opinions of the caretakers and VHTs in the sub-counties that are hard hit by malnutrition and key stakeholders in the nutrition programme of Buhweju district.

## Conclusions

Persistent malnutrition in under five children is mainly due to historical and geographical challenges and its associated factors that include poverty and economic occupation, parental alcoholism and domestic violence and inadequate childcare services. Thus literacy education for mothers and young adolescent boys and girls through engaging local leaders, local nongovernmental organizations and Companies operating in the district to contribute to social services provision would limit the domestic violence and increase sensitization on male responsibilities in the children care in Buhweju district.

### Perspective and recommendation

We recommend effective policy changes and enacting and implementing by laws and strengthening multisectoral collaboration between infrastructure, Agriculture, political will and health and community engagement in Buhweju district.

## Supplementary Information

**Additional file 1.**

## Data Availability

To ensure confidentiality of participants’ information as agreed up on during Ethical approval and Consent Process, qualitative interview transcripts and the corresponding anonymised Atlas ti7.5 file are only visible to the direct research team, and are not publically available.

## Abbreviations

C-IMCI: Caregiver knowledge of best practices in terms of Integrated Management of Childhood Illnesses
DHO: District Health Officer
FGD: Focus Group Discussion
HIV: Human Immune Deficiency Virus
KII: Key Informant Interviews
MUST: Mbarara University of Science and Technology
SAM: Severe Acute Malnutrition
SDGs: Sustainable Development Goals
VHT: Village Health Team
WHO: World Health Organization
