# Supplementary material for: Healthcare providers and caregivers’ perspectives on factors responsible for persistent malnutrition of under 5 children in Buhweju district, South Western Uganda; a phenomenological qualitative study
Source: BMC Public Health. 2021 Aug 3;21:1495. doi: 10.1186/s12889-021-11432-1 (PMC8330056; doi:10.1186/s12889-021-11432-1)
Supplement: Supplementary file 1 — Additional file 1. [file 12889_2021_11432_MOESM1_ESM.docx]

**Interview Guides**

**Interview ID…………..**

**Date of Interview…………………**

**Place of Discussion……………...**

**Description of the Key informant………………………………………………………..**

**Comments of how the interview went………………………………….............................**

You have been selected to participate in this interview because of your immense experience and knowledge of the nutrition program, policy and implementation. Since you have been involved in the running of the nutrition program meant to improve the nutrition status of the population in Buhweju district. We expect you to aware of the magnitude of the problem, most affected areas and what the community is doing about the problem.

**QUESTIONS**

1. What is your role in the programs to improve the nutrition status of the population in Buhweju district? (*Ask for real activities, what is your expected output, what are your achievements so far? Do you think there are still malnourished children in those communities*?
2. What are the factors responsible for malnutrition of under five in Engaju and Nyakishana sub-counties of Buhweju district?(*Family factors, community factors, Health care factors, Economic factors, Cultural factors and political factors*)
3. What have people done, at family level to improve child nutrition in Engaju and Nyakishana sub-counties of Buhweju district? (*Primary, Secondary, Tertiary factors*)

**THANK YOU SO MUCH FOR YOUR TIME**

**Interview ID…………..**

**Date of Interview…………………**

**Place of Discussion……………...**

**Description of the Focus Group Discussion ……………………………………………….**

**Comments of how the interview went………………………………………………………**

You have been selected to participate in this Focus Group Discussion because you are stakeholders in the nutrition of the under five children. You are also directly responsible the nourishment of this children and their overall wellbeing. We would like to ask you how you have been taking care of the wellbeing of these children, the challenges encountered and what you think are the possible ways to overcome the challenges.

**QUESTIONS**

1. Do you think under five children need any nutrition care? (Why? Whynot?)
2. What are the factors responsible for malnutrition of under five in Engaju and Nyakishana sub-counties of Buhweju district?(*Family factors, community factors, Health care factors, Economic factors, Cultural factors and political factors*)
3. What have people done, at family level to improve child nutrition in Engaju and Nyakishana sub-counties of Buhweju district? (*Primary, Secondary, Tertiary factors*)

**THANK YOU SO MUCH FOR YOUR TIME**

**Interview ID…………..**

**Date of Interview…………………**

**Place of Discussion……………...**

**Description of the Focus Group Discussion (VHT)………………………………………...**

**Comments of how the interview went……………………………..…………………………**

You have been selected to participate in this Focus Group Discussion because you are members of the Village Health Teams in Buhweju. And you have been participating in a number of activities aiming at community health promotion. Throughout this discussion we shall ask you questions about your experiences in promoting Health of under five children to prevent malnutrition.

**QUESTIONS**

1. Have you come across any malnourished children in your area? (What was the problem and What did you do?)
2. What are the factors responsible for malnutrition of under five in Engaju and Nyakishana sub-counties of Buhweju district? (*Family factors, community factors, Health care factors, Economic factors, Cultural factors and political factors*)
3. What have people done, at family level to improve child nutrition in Engaju and Nyakishana sub-counties of Buhweju district? (*Primary, Secondary, Tertiary factors*)

**THANK YOU SO MUCH FOR YOUR TIME**
